# Assessing Loss of Regulatory Divergence, Genome–Transcriptome Incongruence, and Preferential Expression Switching in Abaca × Banana Backcrosses

**DOI:** 10.3390/genes13081396

**Published:** 2022-08-06

**Authors:** Nelzo C. Ereful, Antonio G. Lalusin, Antonio C. Laurena

**Affiliations:** 1 Biochemistry Laboratory–Plant Physiology Laboratory, Institute of Plant Breeding, College of Agriculture and Food Science, University of the Philippines Los Baños, Laguna 4031, Philippines; 2 Philippine Genome Center for Agriculture, University of the Philippines Los Baños, Laguna 4031, Philippines; 3 Institute of Crop Science, College of Agriculture and Food Science, University of the Philippines Los Baños, Laguna 4031, Philippines

**Keywords:** abaca (*M. textilis*), allelic imbalance, regulatory divergence, banana (*M. balbisiana*), allele-specific expression

## Abstract

The *Musa textilis* var. Abuab has high fiber quality (FQ) but is susceptible to abaca bunchy top virus (AbBTV); the *Musa balbisiana* var. Pacol has low FQ but is resistant against AbBTV. Their backcrosses (BC_2_ and BC_3_) possess both desirable traits. Analysis using RNA-seq showed that the regulatory divergence of Abuab and Pacol is largely explained by cis differences with 27.4% and 22.3% if we are to assess it using BC_2_ and BC_3_, respectively. Cis differences between the two genotypes are significantly reduced from BC_2_ to BC_3_ due to changes in genomic constitution. Trans, on the other hand, is robust to changes in allelic composition. All these are attributed to the loss of heterozygosity in BC_3_ relative to BC_2_. Further analysis showed that both backcrosses exhibited genome-wide preferential expression of Pacol- over Abuab-specific alleles, despite the wider genetic presence of the latter in the hybrids. The ratio of the two genotype-specific expressed transcripts and the ratio of their corresponding genetic make-up are significantly disproportionate, a phenomenon that we refer to here as “genome–transcriptome incongruence”. We also observed preferential expression switching in which several genes prefer the Abuab- (or Pacol-) specific allele in BC_2_ but switched to the Pacol- (or Abuab-) specific allele in the BC_3_ genome.

## 1. Introduction

Abaca (*M. textilis* Née), also known as Manila hemp in the international community, is a close relative of banana. It is widely cultivated in the Philippines, which supplies 85% of the world’s demand [1]. Ecuador, Costa Rica, and other Southeast Asian countries such as Indonesia also supply abaca fiber, although at lower proportions.

This crop is mainly cultivated for its fiber which is used to make ropes, currency notes, and textiles, among others. Its uses have further broadened in the automotive and aerospace engineering industries due to its high tensile strength. In cars, it is used as underfloor protection [2]. Recently, due to the COVID19 pandemic, its use has expanded in the medical industry to fabricate personal protective equipment (PPE) owing to its high medical grade quality [3].

Abaca (T genome) is placed under the Callimusa section, members of which have a ploidy of 2n = 20. *M. acuminata* (A genome), along with *M. balbisiana* (B genome), are diploid but are generally triploid. The double haploid Pahang belonging to *M. acuminata* subspecies *malaccensis* has a ploidy of 2n = 22 with a 523-megabase genome [4]. Varieties with various ploidy (diploid, triploid, and tetraploid) resulted from crossing these A- and B-genomes. The majority of cultivated banana varieties are predominantly triploid [5].

Despite the importance of this crop, studies on its molecular biology are relatively lagging compared to other fiber plants such as jute, cotton, and hemp. The genome sequence of abaca (var. Abuab) has been recently decoded using high-throughput sequencing [6]. This will immensely advance our understanding of its molecular sequence and functional divergence to other *Musa* spp. and will aid breeders in its improvement. A recent paper revealed the high genetic diversity of the abaca germplasm, with Shannon diversity index I = 0.68, using 150 accessions across the Philippines [7].

In this paper, we assayed the parental genotypes abaca var. Abuab and wild banana (*M. balbisiana*) var. Pacol and their backcross hybrids (BC_2_ and BC_3_) for allele-specific expression (ASE) imbalance using RNA-seq. This happens when, in F_1_ hybrids, one of the two alleles is driven by significantly higher expression levels compared to the other allele. F_1_ hybrids are host to two coresiding genomes. Asymmetric expression is attributed to the cis-regulatory divergence between these two specific alleles.

ASE has been a subject of a number of papers on different organisms such as humans [8,9], *Drosophila* [10,11,12], maize [13], stickleback [14], coffee [15], and rice [16,17]. In maize [13], assessment of asymmetric expression showed the prevalence of cis-acting regulatory variations and slight expression bias towards the maternal parent. In coffee, the compatibility of the gene regulatory network depends on the genetic divergence of the two interspecifically divergent parents (*Coffea canephora* and *Coffea eugenioides*) [15]. In rice, arguably the world’s most important crop, assessment of regulatory divergence between non- and water-stress conditions reveals enhancement of cis-/trans-regulatory differences when parental genotypes (IR64 and Apo) and their hybrid were exposed to drought conditions [16]. The study further revealed the association of cis- and/or trans-regulatory divergence to environmental conditions, the latter with heterosis, suggesting the malleability of heterosis to external factors.

There are several approaches to assessing ASE, including single-base extension [18], pyrosequencing [10], and most recently RNA-seq (e.g., [12]). By comparing ASE in the hybrids and the relative expression level of the same gene(s) in the parents, cis- and/or trans-regulatory divergence can be estimated. This approach has been used extensively to estimate the contributions of cis- and/or trans-regulatory factor(s) on the evolutionary divergence of species of *Drosophila* [10,11,12], stickleback [14], and coffee [15]. A separate study by [19] showed the asymmetric expression of stay-green 1, a key chlorophyll degradation gene, in the banana AAB/ABB cultivars.

Allelic imbalance is mostly assayed in F_1_ hybrids. However, there are several studies that used introgression lines such as monkeyflower [20], *Drosophila* [21], mouse [22], and *Solanum* [23] to assess ASE imbalance. In monkeyflower (*Mimulus guttatus* and *Mimulus nasutus*), for example, an examination of ASE showed that hybrid sterility is explained by rampant misexpression and not by regulatory divergence [20]. On the other hand, contrary to previous reports that cis largely explains regulatory divergence, a report on *Solanum* (*Solanum pennellii* and *Solanum lycopersicum*) showed that cis- and trans-regulatory factors equally contribute to evolutionary divergence [23].

In this study, we sequenced the total transcript population of both backcrosses, including their parents using RNA-seq, to elucidate ASE imbalance in the backcrosses and the regulatory divergence in the parental genotypes. To our knowledge, no study has been performed to assess genome-wide allelic imbalance in *Musa* spp. hybrids, moreso in backcrosses; thus, this investigation to evaluate this imbalance.

## 2. Materials and Methods

**Figure 1:**
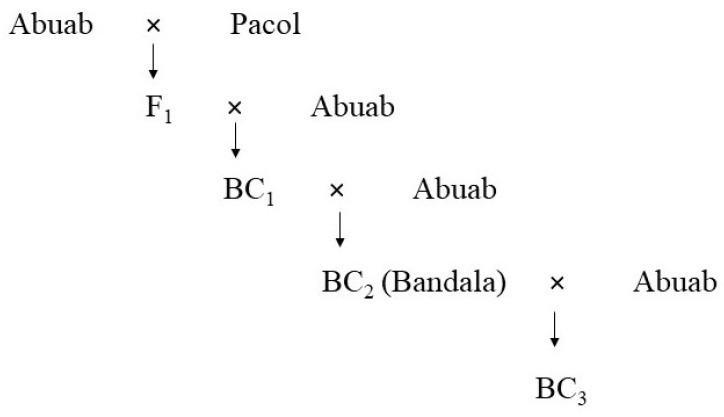
F_1_BC_2_ (Bandala) and F_1_BC_3_ were created by crossing Abuab and Pacol. Abuab serves as the maternal parent; Pacol as the pollen donor at F_0_. In the succeeding generations, Abuab serves as the pollen donor. Note that BC_2_ is locally named as “Bandala.”.

The abaca (*M. textilis*) var. Abuab, the wild banana (*M. balbisiana*) var. Pacol and their backcrosses, BC_2_ and BC_3_, were grown and maintained at Feeds and Industrial Crops Section (FICS) collection site of the Institute of Plant Breeding (IPB), UPLB (14°09′09.7″ N 121°15′39.2″ E). A schematic diagram is shown on how backcrosses were generated (Figure 1).

### 2.1. RNA Extraction

The central whorls of the stalk samples of three-month-old suckers were collected on 22 June, 2021, between 10:00 a.m. and 11:00 a.m. Samples were snap-frozen in liquid N and were stored in a −80 °C freezer until further extraction.

Three replicates of the same variety were pooled in equal weight (~0.33 g) to make up to 1.0 g. This was previously referred to as biological averaging [24], which was found to be more cost-efficient while maintaining statistical power [25]. In addition, we preferred pooling of samples due to the difficulty in extracting RNA from abaca and banana pseudostem samples. Pooling of samples for RNA-seq has been performed in recent studies [26,27]. Pooled samples were then ground using mortar and pestles. All autoclavable equipment and reagents were sterilized to avoid unwanted contamination.

We followed the modified cetyl trimethylammonium bromide (CTAB) protocol with the 2% ß-mercaptoethanol method with LiCl precipitation for RNA extraction as previously described by Dr. John Carlson of Schatz Center for Tree Molecular Genetics at Pennsylvania State University. RNA was reconstituted using 800 µL SSTE with 4 uL Monarch^®^ (New England Biolabs, Ipswich, MA, USA) DNAse in 20 uL DNAse buffer, incubated at 37 °C for 1 h. DNAse was removed by adding 24:1 chloroform–isoamyl alcohol. After spinning for 20 min at 14,000 rpm at 4 °C, the aqueous phase was obtained and 0.1 volume of Na acetate and 2 volumes of absolute ethanol was added. Samples were incubated for 1 h at −80 °C, then spun for 30 min at 14,000 rpm at 4 °C. The pellet was obtained and washed with 75% ethanol by spinning. Pellets were dried for 15 min, and 50-uL nuclease-free water was added. Bands were resolved using 2% agarose gels and stained with GelRed^®^. The quality and yield of total RNA were assessed using a BioTek^®^ (BioTek Instruments Inc., Winooski, VT, USA) Epoch Microplate Spectrophotometer controlled with Gen5 software.

The RNA samples were sent to Macrogen Korea (Seoul, Korea) for RNA sequencing (with rRNA depletion, Ribo-Zero) using Illumina Novaseq 6000, paired-end (PE). Library preparation and sequencing procedures were performed according to the protocol followed by the company.

### 2.2. Bioinformatics Analysis

Statistical analysis was performed using Rstudio v.1.4.1717 [28], while the computational pipeline used the Linux environment (Debian 4.9.246-2). All R scripts and Linux commands are stored in our GitHub repository site: https://bit.ly/3tP4BtE (accessed on 12 June 2022).

#### 2.2.1. Reads Preprocessing

Reads were quality checked using FastQC [29]. No adapters and low-quality reads were found; therefore, no further preprocessing step was performed.

#### 2.2.2. Mapping

The reads generated from the parental genotypes *M. textilis* var. Abuab and *M. balbisiana* var. Pacol were aligned against the genome references Abuab [6] and double haploid of Pisang Klutuk Wulung (DH–PKW) [5], respectively, using STAR v.2.7.7a [30] with the following arguments: --outFilterMatchNmin 0, --outFilterScoreMinOverLread 0.3, --outFilterMatchNminOverLread 0.3. Because Abuab (*M. textilis*) and Pacol (*M. balbisiana*) are interspecifically divergent, we searched for transcript orthologs using OrthoVenn2 [31] with the protein fasta sequence of both reference assemblies as inputs, implementing an E-value of 1 × 10^−5^ and inflation index of 1.5.

To assess regulatory divergence between the parents and ASE in the backcrosses, we followed the pipeline as previously described [20,32,33] with modifications. Reads from BC_2_ and BC_3_ genotypes were competitively aligned against the concatenated Abuab and DH–PKW genome reference assemblies, also using STAR, with the same parameters as described above.

#### 2.2.3. Read Count Quantification and Normalization

For ASE analysis in the backcrosses, uniquely mapping reads aligning to each reference assembly were quantified using the subread featureCount function [34]. We counted the primary alignment alone (option: --primary) with the following additional options: -t exon, the feature type to count read against, and; -g transcript_id, the attribute type to summarize counts. These unique reads aligning to orthologous transcripts of merged pseudoreference assemblies represent ASE [33]. A raw data of read counts corresponding to the various genotypic counts of the parents and allele-specific counts from the backcrosses (columns) and transcript orthologs (rows) was created.

Reads with a total row sum of 0 were removed to increase computational speed and reduce data size. We normalized the reads using edgeR’s Trimmed Means of M values with lowly expressed genes being filtered out [35]. The total normalized read counts between the parents were calculated. The total should be at least 20 (Abuab + Pacol ≥ 20) to be considered for further analysis. We added 1 for read counts with 0 values to avoid a 0 numerator or denominator; the sum, however, should be at least 20. The expression ratios and their log(2) fold-change(FC) between the two parental read counts and the binomial exact test of equal proportion (*p* = 0.5, with FDR-adjusted *p*-values; in R) were calculated. Any significant difference between the parental orthologous transcripts gives estimates of the parental expression divergence (P). Likewise, in the BC_2_ and BC_3_ genotypes, the log(2)FC between the two genotype-specific alleles (H) in each backcross and their binomial exact test (*p* = 0.5; using R) were calculated (see GitHub repository for custom R scripts we created).

Trans effects were estimated by subtracting H from P (T = P − H) [11,36,37]. We then tested the datasets using Fisher exact test followed by FDR analysis.

For cis- and/or trans-regulatory assignments (cis, trans, cis × trans, cis – trans, and cis + trans), we considered the most conservative analysis (i.e., binomial and Fisher’s exact tests, FDR < 0.5%) in the Results and Discussion (Section 3) as previously performed (e.g., [12,17]). For cis-/trans-regulatory assignments, see our GitHub repository site: https://bit.ly/3tP4BtE (accessed on 6 May 2022).

#### 2.2.4. Allelic Imbalance Polymorphism in the Backcrosses

To assess expression imbalance between heterozygous sites (i.e., orthologous genes between the two genotype-specific alleles) in BC_2_ and BC_3_, we implemented a binomial exact test, with a null hypothesis of equal proportion (*p* = 0.50). We used a custom R script to execute this command (see GitHub site). Transcript orthologs that exhibit an FDR < 0.05 are said to be significantly asymmetrically expressed. Additionally, we calculated the log-transformed expression ratios of the read counts (Abuab-/Pacol-specific allele) to the base 2 (Log_2_FC). A biological threshold of |log (2) FC| ≥ 1, binomial (*p* = 0.5, FDR < 0.05) was implemented. Only features with a total normalized read count of 20 reads between the two specific alleles were considered for further analysis (Abuab_BC2_ + Pacol_BC2_ ≥ 20; Abuab_BC3_ + Pacol_BC3_ ≥ 20) to ensure genes are expressed and to avoid inclusion of artifacts, as previously performed (e.g., [12,16,37,38]). We incremented read counts with a value of 1 for genotype-specific alleles with 0 read counts to avoid a numerator or a denominator of 0.

We also tested the proportions between parental genotypes and genotype-specific alleles using a z-score test for two population proportions, two-tailed, with *N* = 33,511 (for BC_2_) and *N* = 33,394 (BC_3_). These values correspond to the combined estimated numbers of the predicted genes for *M. textilis* Abuab (33,277) and *M. balbisiana* DH–PKW (35,148) [6].

#### 2.2.5. Relative Transcript Accumulation Ratio in the Backcrosses

We concatenated the normalized read counts of both BC_2_ and BC_3_ into a single matrix. The sum of the normalized read counts of the genotype-specific alleles in each backcross was calculated. Only transcript orthologs with a total normalized read count of 20 in either or both backcrosses were considered for further analysis. As performed above, we added 1 for specific alleles with 0 values. Expression ratios between the two genotype-specific alleles (Abuab/Pacol) were log(2)-transformed in both hybrids. Genes that are asymmetrically expressed at a biological threshold of |Log_2_FC| ≥ 1 and statistical threshold of FDR < 0.5% (binomial test of equal proportion, *p* = 0.5) in either or both genotype-specific alleles were considered for further classification. Genes that exhibit FDR < 0.5% are said to be asymmetrically expressed.

Transcript orthologs that are not significantly differentially expressed using a binomial exact test (FDR < 0.5%) in both backcrosses are said to exhibit no ASE imbalance.

#### 2.2.6. Gene Ontology (GO) Enrichment Analysis

Functional annotations of the genes were taken from the annotation provided for the Abuab reference sequence [6]. GOfuncR (hypergeometric test, overrepresented at FWER < 0.1) [39] was used for GO functional enrichment analysis. Unless otherwise indicated, we used 0.1 as a statistical threshold for most of the GO enrichment analyses since FWER provides a conservative measure of error rate. The participation of these genes in the predicted pathways was further confirmed in published literature.

## 3. Results and Discussion

The abaca (*M. textilis*) var. Abuab was found to have high fiber quality but is susceptible to AbBTV [40]. On the other hand, Pacol, an *M. balbisiana* variety, possesses phenotypic characters contrasting to that of Abuab, i.e., low fiber quality but resistant against AbBTV [41,42,43]. These two *Musa* spp. were crossed to create the F_1_, which was successively crossed to Abuab to create the backcrosses BC_2_ and BC_3_ (see Figure 1) to identify allelic imbalance polymorphism and map cis- and/or trans-regulatory divergence.

Pacol is a diploid and was shown to have a chromosome number of 2n = 22, while Abuab has 2n = 10. Selection of F_1_ and BC_1_ individuals with abaca-like phenotypic traits and successive backcrossing of the selected progenies to Abuab showed a ploidy level of 2n = 20 for both BC_2_ and BC_3_ based on morphological characterization and karyotyping analysis [42].

We chose backcrosses (not the F_1_ hybrids) for allelic imbalance assay, as these are economically important genotypes with the desirable traits of industrial significance—superior FQ and AbBTV resistance. Recent studies on several organisms showed that asymmetrically expressed genes were found associated with traits of interest, including rice [44], yeast [45], and banana [19]. Therefore, it would be interesting to identify asymmetrically expressed genes, as they could be potential candidates for the traits of interest.

BC_2_ or Bandala has been planted in multilocation trial sites in the different regions of the Philippines: Luzon, Visayas, and Mindanao [46,47]. BC_3_, on the other hand, was relatively recently developed. Based on an initial field assessment, it has superior fiber quality and is resistant against abaca bunchy top virus (AbBTV). A molecular study using RNA-seq revealed that it is the best among five abaca genotypes, as it induces a significantly higher number of genes associated with both desirable traits [16].

**Figure 2:**
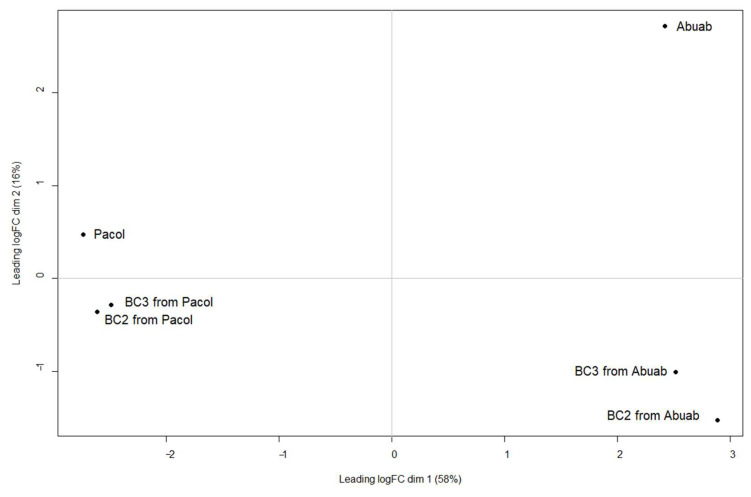
MDS plot of the parental genotypes and the genotype-specific alleles derived from each backcross. Legend: “BC_2_ from Pacol” and “BC_3_ from Pacol” indicate the total population of transcripts derived from Pacol in the BC_2_ and BC_3_ genomes, respectively. “BC_2_ from Abuab” and “BC_3_ from Abuab” indicate the total population of transcripts derived from Abuab in the BC_2_ and BC_3_ genomes, respectively.

We generated multidimensional scaling (MDS) plots using the normalized read counts to visualize the relationship among the genotypes and each coresiding genome in the backcrosses. Results (Figure 2) clearly indicate that Dim. 1 largely explains the divergence of the two genotypes with Abuab and Pacol and their respective backcrosses clearly separated. Dim. 2, on the other hand, separates the parental genotypes from the backcrosses.

### 3.1. Regulatory Divergence between Abuab and Pacol

The genome-wide expression performance of the abaca-specific genes of BC_2_ and BC_3_, along with their parents, was shown in a companion paper [16]. However, assessment of regulatory divergence between the parents and asymmetric expression in their backcrosses has not been performed.

Briefly, we aligned the parental reads against the Abuab reference sequence [6] for Abuab and DH–PKW [5] for Pacol. The backcross reads, on the other hand, were mapped against the concatenated reference assemblies (see Materials and Methods (Section 2)). Only the uniquely mapping reads were quantified. Transcripts with corresponding orthologs between the two genotype-specific alleles with a total normalized read count of 20 were retained for further analysis (unmatched transcripts were dropped as previously performed [48]).

Results showed that there were 14,868 transcript orthologs or transcripts with matching sequences between the two *Musa* spp. peptide reference assemblies. This accounts for nearly 50% of the transcript population of both varieties, suggestive of their degree of expression divergence.

**Figure 3:**
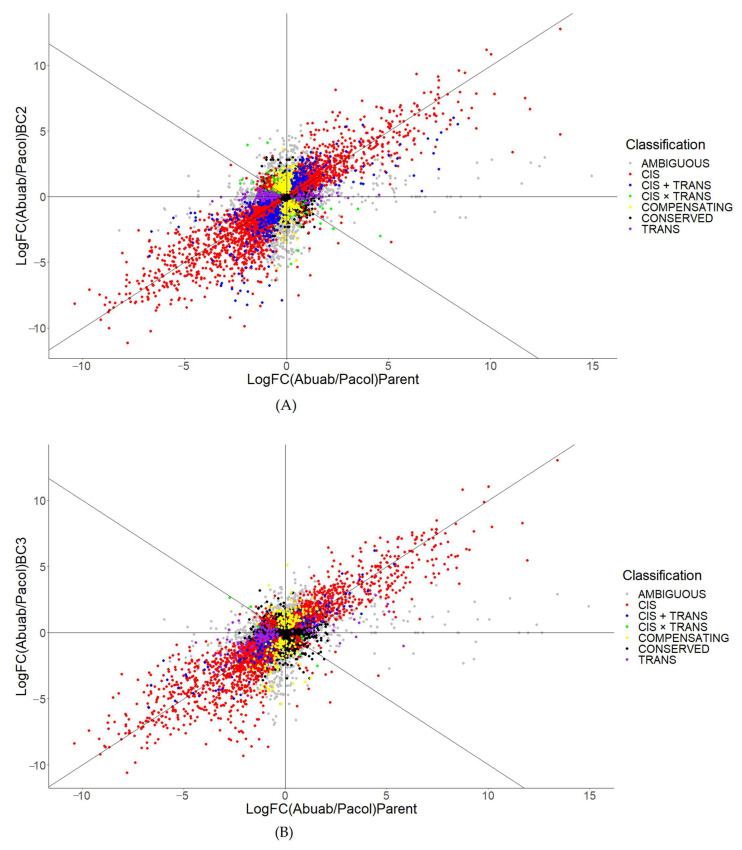
The cis- and/or trans-regulatory divergence architecture between Abuab and Pacol as estimated using their (**A**) BC_2_ and (**B**) BC_3_ backcross hybrids. Figure generated using ggplot2 [52] in Rstudio [28].

Regulatory differences between intra- or interspecifically related organisms are often estimated using their F_1_ progenies. In our case, we used backcross hybrids to estimate regulatory divergence between the two interspecifically divergent *Musa* spp. We found 9295 genes with read counts greater than 20 between the parents (Abuab + Pacol ≥ 20). Results indicate that regulatory divergence between Abuab and Pacol is largely explained by cis differences with 27.4% and 22.3% as estimated and assayed in BC_2_ and BC_3_, respectively (excludes ambiguous and conserved; Table 1; red points in Figure 3; see also Tables S1 and S2 for lists of orthologous genes diverging in cis and/or trans using BC_2_ and BC_3_, respectively). If we are to extrapolate these values, the degree of expression divergence using F_1_ will be mostly explained by cis differences with proportion beyond these values. This, however, is a speculation and is an interesting area of further inquiry. The finding that cis-regulatory factors explain interspecific divergence has been widely reported in the literature (e.g., [10,14,17,49,50]). On the other hand, the trans-regulatory factor (purple in Figure 3) and its interaction with cis (synergistic, antagonistic, and compensating) may also explain their divergence, albeit very modestly.

Further analysis showed that the number of cis-diverging genes is significantly reduced from BC_2_ to BC_3_, while the conserved transcript orthologs are significantly increased (*p* < 0.0001; Table 1). This is attributed to the loss of heterozygosity (or increase in homozygosity) in the BC_3_ genome relative to BC_2_. These results suggest that with the changes in genomic constitution, cis is significantly affected due to the loss of Pacol (or gain of Abuab) genomic segments. The loss or gain of genetic sequences may play roles in the transcriptional regulation of alleles, which contribute to the adaptive evolution of organisms. The interactions of cis with trans are, likewise, significantly affected (synergistic, antagonistic, and compensatory; *p* < 0.0001). Points representing these interactions, including trans, are shown in Figure 3A,B (BC_2_ and BC_3_, respectively).

Trans, on the other hand, is robust to changes in genomic composition (*p* = 0.3817). In recent papers, trans is significantly affected by changes in external factors [17,36] but not in changes in genetic make-up. On the contrary, cis is robust to environmental changes [17,51].

### 3.2. Asymmetric Expression

We wanted to test whether asymmetric expression exists at sites heterozygous between two genotype-specific alleles in the backcross lines. This will shed light on the biased directionality of expression of orthologous genes between the two coresiding genomes.

#### 3.2.1. Allelic Imbalance in the BC_2_ Genome

Briefly, we concatenated and normalized the read counts of the two genotype-specific alleles in the backcrosses. MA plots showed symmetrical data clouds in both BC_2_ and BC_3_ (Figures S1 and S2, respectively), which indicates that normalization was effective. We implemented a binomial exact test on the normalized read counts (at |Log2FC| ≥ 1; *p* = 0.5, FDR < 0.05, see Materials and Methods (Section 2)) to identify genes that are asymmetrically expressed from the expected ratio of 1:1. We found 3971 transcript orthologs that satisfy these criteria, of which 1703 and 2268 preferentially expressed Abuab- and Pacol-specific alleles, respectively, in BC_2_ (Table S3). (Note that these values exclude symmetrically expressed genes. That is, those that do not satisfy both/either biological and/or statistical thresholds).

Therefore, these results revealed that BC_2_, on a transcriptome-wide scale, preferentially expressed the *M. balbisiana* (banana) Pacol over the *M. textilis* (abaca) Abuab allele. This is in contrast to its expected genomic constitution in which BC_2_ theoretically hosts 87.5% Abuab and 12.5% Pacol alleles. There is a significant discrepancy of these proportions to the ratio of the expressed genotype-specific transcripts in BC_2_, 43% Abuab–57% Pacol (z = 70.4, *p* < 0.0001; z-score test for two population proportions). This shows that the genetic ratio of the two alleles in the backcross hybrids and its corresponding transcript expression ratio are significantly disproportionate, a condition which we refer to here as “genome–transcriptome incongruence”.

In our previous work on the asymmetric expression of rice [44], there was 41–59% expression of Apo- and IR64-specific alleles in the F1 transcriptome. At the genome level, we expect a 50–50% contribution of the parental genotypes. It is now increasingly clear that there appears to be a significant incongruence between genome and transcriptome ratios.

In the BC_2_ nucleus, both parent-specific alleles are exposed to the same trans-regulatory factors. Therefore, allelic imbalance between two heterozygous sites has been ascribed to cis divergence and a varying allele-specific epigenetic landscape [10,11].

As polysaccharide and lignin were found associated with high fiber quality [16], we searched for such genes allelically imbalanced in BC_2_. We found 16 genes encoding for either putative cellulose synthase or cellulose synthase, 14 of which preferred the Abuab-specific allele (|Log2FC| ≥ 1; FDR < 0.05). Abuab confers the high fiber quality of the backcrosses. Six genes encoding for Cinnamoyl-CoA reductase, an enzyme involved in lignin biosynthesis [53], were found asymmetrically expressed, and three genes preferred each specific allele.

Gene Ontology (GO) analysis using GOfuncR [39] of the Abuab-specific transcript orthologs significantly differentially expressed using a binomial exact test (FDR < 0.05) showed enrichment of genes associated with cellular component biogenesis, cellular component organization or biogenesis, and ribonucleoprotein complex biogenesis (Biological Process, FWER < 0.1) (see Table S4). On the other hand, no GO terms were found enriched in Pacol-specific transcript orthologs (FWER < 0.1) using GOfuncR.

#### 3.2.2. Allelic Imbalance in the BC_3_ Genome

In BC_3_, 3561 were found significantly asymmetrically expressed between the two alleles (|Log2FC| ≥ 1; *p* = 0.5, FDR < 0.05) (Table S5). Of these, 1609 (45%) and 1952 (55%) preferentially expressed the Abuab- and Pacol-specific alleles, respectively. Similar to Bandala (BC_2_), there is a broader expression proportion of the banana allele as compared to the abaca allele. This is contrary to its genetic background in which BC_3_ is made up of 93.75% Abuab and 6.25% Pacol alleles. The expression proportion of the Pacol allele is still significantly large considering its modest genetic presence in BC_3_ (z = 88.30; *p* < 0.00001; z-score test for two population proportions, two-tailed). There appears to be a consistent observation that the genetic ratio between the two alleles and their corresponding transcript expression ratio are incongruent in the backcrosses.

There are 3971 asymmetrically expressed in BC_2_ and only 3561 in the BC_3_ genotypic line (FC ≥ 2; *p* = 0.5, FDR < 0.05). This shows that BC_3_ exhibited a lesser number of genes asymmetrically expressed as compared to BC_2_ potentially due to the diminishing or gradual loss of heterozygosity (or increasing homozygosity) as a consequence of backcrossing to the recurring parent. This has been partly observed in a previous paper by [54], which states that the loss of heterozygosity, a common form of allelic imbalance, happens when heterozygotic lines become homozygous because one of the two alleles gets lost.

Fifteen (15) genes encoding for either cellulose synthase or putative cellulose synthase were found to exhibit bias expression, mostly preferring the Abuab allele (14 genes), with one gene preferring the Pacol-specific allele (FC ≥ 2; FDR < 0.05). Notably, a gene encoding for Photosystem I assembly protein was the most allelically imbalanced gene, exhibiting 13-fold and preferentially expressing the Abuab-specific allele. This suggests an active photosynthetic activity during fiber synthesis at the vegetative stage with preferential expression favoring the Abuab allele. No GO terms were found enriched in either of the specific alleles (at FWER < 0.1) using GOfuncR.

#### 3.2.3. Extreme Cases of Allelic Imbalance

Genes encoding for Photosystem I assembly protein (LOCUS_023583-RA; 13× log(2)FC), pre-mRNA-processing-splicing factor 8A (LOCUS_012301-RA; 11×), serine carboxypeptidase-like 50 (LOCUS_015865-RA; 11×), and ABC transporter G family member 31 (LOCUS_002982-RA; 9.5×) were observed to exhibit the highest expression Log_2_FC, all preferring the Abuab-specific allele in BC_2_ (Table S2). Interestingly, the same genes were found to exhibit extremely high fold expression in BC_3_ (Table S3), with the Abuab allele being favored: Photosystem I assembly protein (LOCUS_023583-RA; 13.6×), ABC transporter G family member 31 (9.4×), pre-mRNA-processing-splicing factor 8A (LOCUS_012301-RA; 11.6×), and serine carboxypeptidase-like 50 (LOCUS_015865-RA; 9.9×) (note: all fold expression values are in Log_2_FC). These findings highlight the active engagement of photosynthesis, transcription, and transport during the synthesis of fiber components (sugars and lignin) in both backcrosses, with the Abuab-specific allele being preferred over the Pacol-specific allele.

The Pacol allele, on the other hand, confers the abaca bunchy top virus (AbBTV) resistance. We found four genes (Mba11_g21030, Mba05_g06300, Mba06_g30590, and Mba04_g19640) encoding for (Enhanced) Disease Resistance protein favoring the Pacol allele commonly asymmetrically expressed in both backcrosses (note: because Pacol is an *M. balbisiana* var., we used the DH–PKW naming system). Because the genotypes were not infected, genes associated with disease resistance are modestly detected. Challenging the genotypes with AbBTV is highly recommended in succeeding studies to identify allelically imbalanced genes under biotic stress.

**Figure 4:**
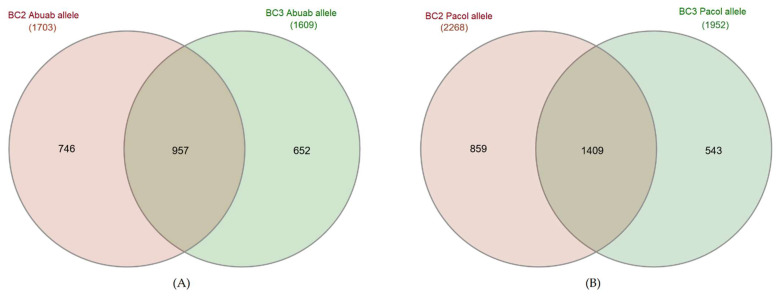
Number of genes commonly and uniquely asymmetrically expressed preferring (**A**) Abuab-specific allele and (**B**) Pacol-specific alleles between the two backcrosses.

Furthermore, we found 957 genes commonly preferring the Abuab-specific allele in both backcrosses and 1409 genes preferring the Pacol-specific allele (Figure 4). These results suggest the consistency of a specific allele being preferentially expressed.

### 3.3. Relative Transcript Accumulation Ratio in the Backcrosses

Above, we showed that cis differences explained the divergence between the two parents and the prevalence of allelic imbalance polymorphism in each backcross. To assess changes in the preferential expression between alleles, the relative Abuab/Pacol log-transformed accumulation ratios in these backcrosses were analyzed. Figure 2 (Table S6) depicts the overall cis differences attributed to the variations in the genetic segments between the two backcrosses. Differences in the magnitude (level of expression) and direction (preference) are ascribed to cis differences with the addition of Abuab segments (loss of Pacol segments) acquired by BC_3_ as a consequence of further backcrossing BC_2_ to the recurring parent.

**Figure 5:**
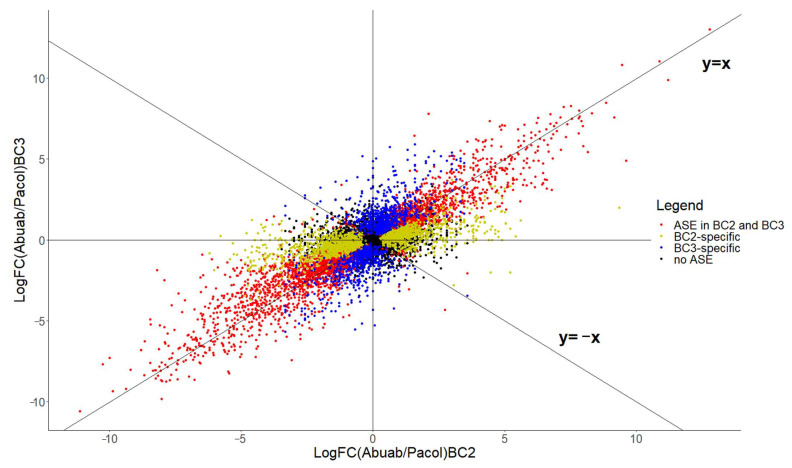
The overall cis differences between BC_2_ and BC_3_ (discussed in the text). The relative abundance of transcript isoforms (expressed as log-transformed Abuab/Pacol ratios) exhibiting ASE imbalance in BC_2_ alone (blue), BC_3_ alone (yellow), and both (red). Black points are genes exhibiting no ASE imbalance. Figure generated using ggplot2 (https://ggplot2.tidyverse.org (accessed on 16 March 2022); [52]) in Rstudio [28].

Results indicate that there are 10,112 transcript orthologs in either/both backcrosses that have more than 20 total normalized read counts. Of these, 2980 genes (29.5%) exhibit commonly asymmetrically expressed between the backcrosses signifying common cis-regulatory differences (red dots lying closely in the y = x curve in Figure 1; binomial exact test of equal proportion, FDR < 0.5%). These are genes exhibiting conserved cis differences between the two hybrids with significant asymmetric expression (termed here as “conserved asymmetric”). GO enrichment analysis showed genes to be significantly associated with intracellular transport, cellular localization, and establishment of localization in cell (Biological Process, BP) and purine ribonucleoside triphosphate binding, and ATP binding (molecular function, MF, FWER < 0.05) (Table S7). This shows that these genes are involved during transport and binding of a range of molecules. On the other hand, 4329 (42.8%) did not exhibit asymmetric expression (no ASE; black dots in Figure 5). This proportion suggests that an enormous number of the total transcript population are symmetrically expressed in the backcrosses; hence, “conserved symmetric”.

There were 1576 (15.6%) genes allelically imbalanced in BC_2_ that were symmetrically expressed in BC_3_, i.e., BC_2_-specific ASE imbalance (yellow dots in Figure 5). On the other hand, there were 1227 (12.1%) genes exhibiting asymmetric expression in BC_3_ that were symmetrically expressed in BC_2_; i.e., BC_3_-specific ASE (blue dots in Figure 5). Apparently, there is a reduction of biased directionality of ASE from BC_2_ to BC_3_. These changes are ascribed to the augmentation or further “dilution” of Abuab segments (or loss of Pacol allele) in the BC_3_ genome, as well as the reduction of heterozygous (or increase in homozygous) cis-regulatory elements. Increasing homozygosity means decreasing cis differences.

We expect that all asymmetrically expressed genes in both BC_2_ and BC_3_ (red dots) should lie on the y = x curve. However, there are also genes allelically imbalanced in both backcrosses lying on the y = −x curve (red points). These genes exhibit preferential expression switching (or allelic imbalance switching) in which a particular gene prefers the Abuab- (or Pacol-) specific allele in BC_2_ but switches to the Pacol- (or Abuab-) specific allele in BC_3_, or vice-versa. There are 59 genes (FDR < 0.5%) that exhibit this kind of swinging expression behavior and are significantly enriched in protein domain-specific binding (MF, GO:0019904, FWER < 0.05; Table S5). There were 34 genes preferentially expressing the Pacol- over the Abuab-specific allele in BC_2_ but then preferred Abuab over the Pacol allele in BC_3_ (FDR < 0.5%). On the contrary, there were 25 genes preferentially expressing Abuab over the Pacol allele in BC_2_ but switched vice-versa in BC_3_. This phenomenon demonstrates the transcriptional versatility of advanced backcross genotypes owing to heterozygosity.

Preferential expression switching has been reported previously, however, between contrasting water regimens, i.e., non- and water-stress conditions in rice [17]. In the current study, preferential expression switching happens between two backcrosses.

## 4. Conclusions

Backcross hybrids (BC_2_ and BC_3_) from *M. textilis* (abaca var. Abuab) × *M. balbisiana* (wild banana var. Pacol) were created to assess cis- and/or trans-regulatory divergence between the parents. We, likewise, interrogated whether there exists asymmetric expression at sites heterozygous to these backcross hybrid lines. Abuab has high FQ but has low resistance against AbBTV; Pacol has low FQ but is resistant against AbBTV. Previous studies showed that their backcrosses possess both desirable traits. Using RNA-seq, results indicated that both backcrosses exhibited genome-wide preferential expression of Pacol- over Abuab-specific alleles, despite the wider genetic presence of the latter in the hybrids: 87.5% and 93.75% in BC_2_ and BC_3_, respectively. We call such an observation “genome–transcriptome incongruence” in which the ratio of the two genotype-specific expressed transcripts and the ratios of their corresponding genetic make-up are significantly disproportionate. Further analysis showed that regulatory divergence of Abuab and Pacol is largely explained by cis differences with 27.4% and 22.3% if we are to assess it using BC_2_ and BC_3_, respectively. The evolutionary divergence of the two interspecifically divergent genotypes is mostly explained by cis differences, and we speculate that it would be higher than these proportions if we are to estimate it using F_1_. We further identified asymmetrically expressed genes associated with high fiber quality and AbBTV resistance. As recent studies revealed, these allelically imbalanced genes may be candidate features for further confirmation tests. Because of the loss of heterozygosity (or increase in homozygosity) in the BC_3_ relative to the BC_2_ genome, cis differences between the two backcrosses are significantly reduced from BC_2_ to BC_3_. This provides clues that cis variations are significantly affected by changes in allelic composition. Trans, on the other hand, is unaffected by changes in allelic composition. Taken together, we have provided preliminary findings consistent with the literature in which hybrids are endowed with transcriptional versatility, which confers them with desirable traits, in this case, high FQ and resistance against AbBTV.

## Figures and Tables

**Table 1:** Number of transcript orthologs exhibiting cis- and/or trans-regulatory divergence between Abuab and Pacol assayed using their BC_2_ and BC_3_. χ^2^ test (with *p*-values, two-tailed; test for 2 × 2 contingency table with Yates’ continuity correction) indicates the significant difference of the number of genes classified in each regulatory category between the two backcrosses.

Regulatory Factors/Interactions	F_1_BC_2_ (%)	F_1_BC_3_ (%)	χ^2^ Stat with Yates Correction	*p*-Value (Two-Tailed)
Cis	2647 (27.4)	2070 (22.3)	66.73	<0.0001
Cis + trans (synergistic)	361 (3.7)	121 (1.3)	112.48	<0.0001
Cis × trans (antagonistic)	478 (5.0)	14 (0.2)	429.55	<0.0001
Compensating	441 (4.6)	203 (2.2)	81.21	<0.0001
Trans	161 (1.7)	139 (1.5)	0.79	0.3736
Ambiguous	2955 (30.6)	3090 (33.2)	15.08	0.0001
Conserved	2613 (27.1)	3658 (39.4)	322.74	<0.0001
Total	9656	9295		

## Data Availability

All datasets have been uploaded in EMBL-EBI ArrayExpress (www.ebi.ac.uk/arrayexpress (accessed on 6 January 2022)) with an assigned accession number: E-MTAB-10990.

## Supplementary Materials

The following supporting information can be downloaded at: https://www.mdpi.com/article/10.3390/genes13081396/s1, Figure S1: MA plot between Abuab- and Pacol-specific alleles in BC_2_ or Bandala; Figure S2: MA plot between Abuab- and Pacol-specific alleles in BC_3_; Table S1: Regulatory divergence between abaca and banana as estimated using BC_2_; Table S2: Regulatory divergence between abaca and banana as estimated using BC_3_; Table S3: Asymmetric expression in BC_2_ using normalized read counts with binomial exact test of equal proportion; Table S4: GO enrichment analysis of allelically imbalanced genes in Abuab allele in BC_2_; Table S5: Asymmetric expression in BC_3_ using normalized read counts with binomial; Table S6: Relative transcript accumulation and preferential expression switching list of genes; Table S7: GO enrichment of genes allelically imbalanced in both BC_2_ and BC_3_.
