# Supplementary figures and images for: Assessing Loss of Regulatory Divergence, Genome–Transcriptome Incongruence, and Preferential Expression Switching in Abaca × Banana Backcrosses
Source: Genes (Basel). 2022 Aug 6;13(8):1396. doi: 10.3390/genes13081396 (PMC9407414; doi:10.3390/genes13081396)

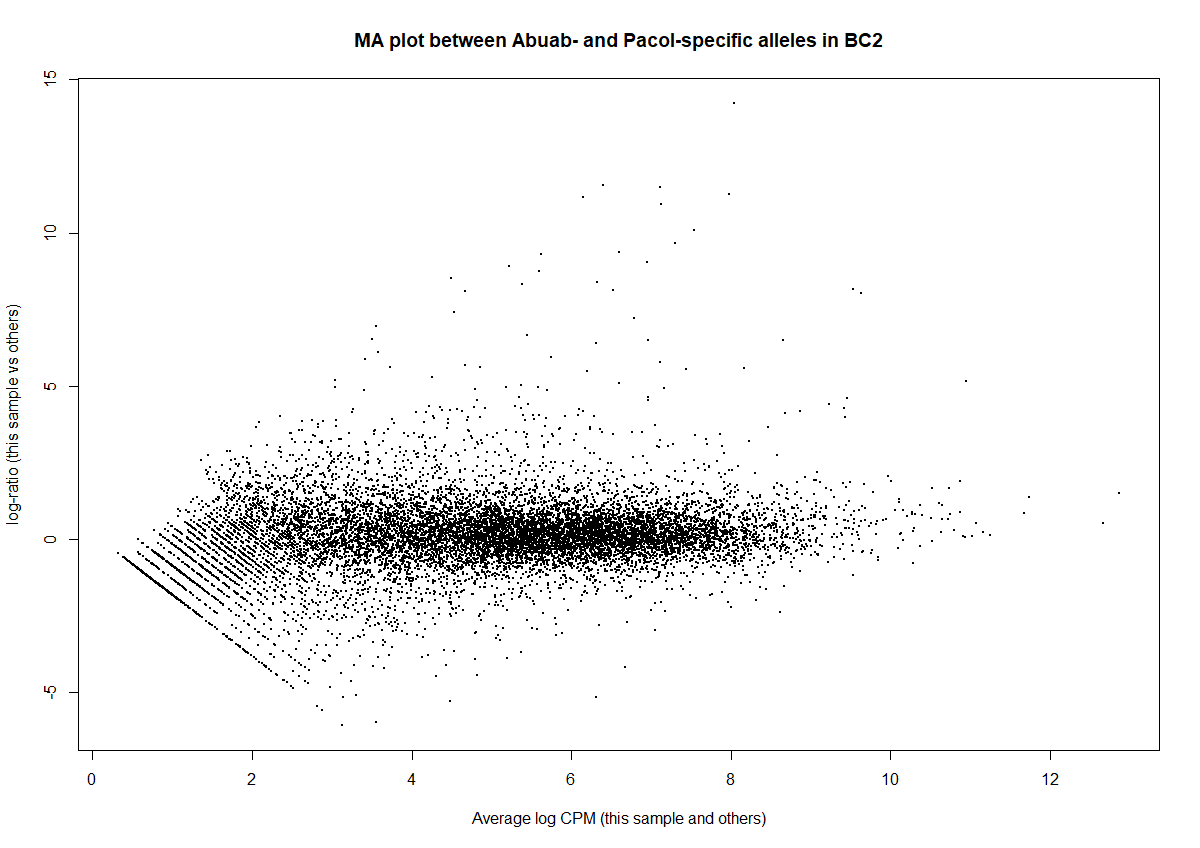

Supplement: Supplementary file 1 [file genes-13-01396-s001.zip › Fig.ure S1. MA plot between Abuab and Pacol specific alleles in BC2 or Bandala.png]

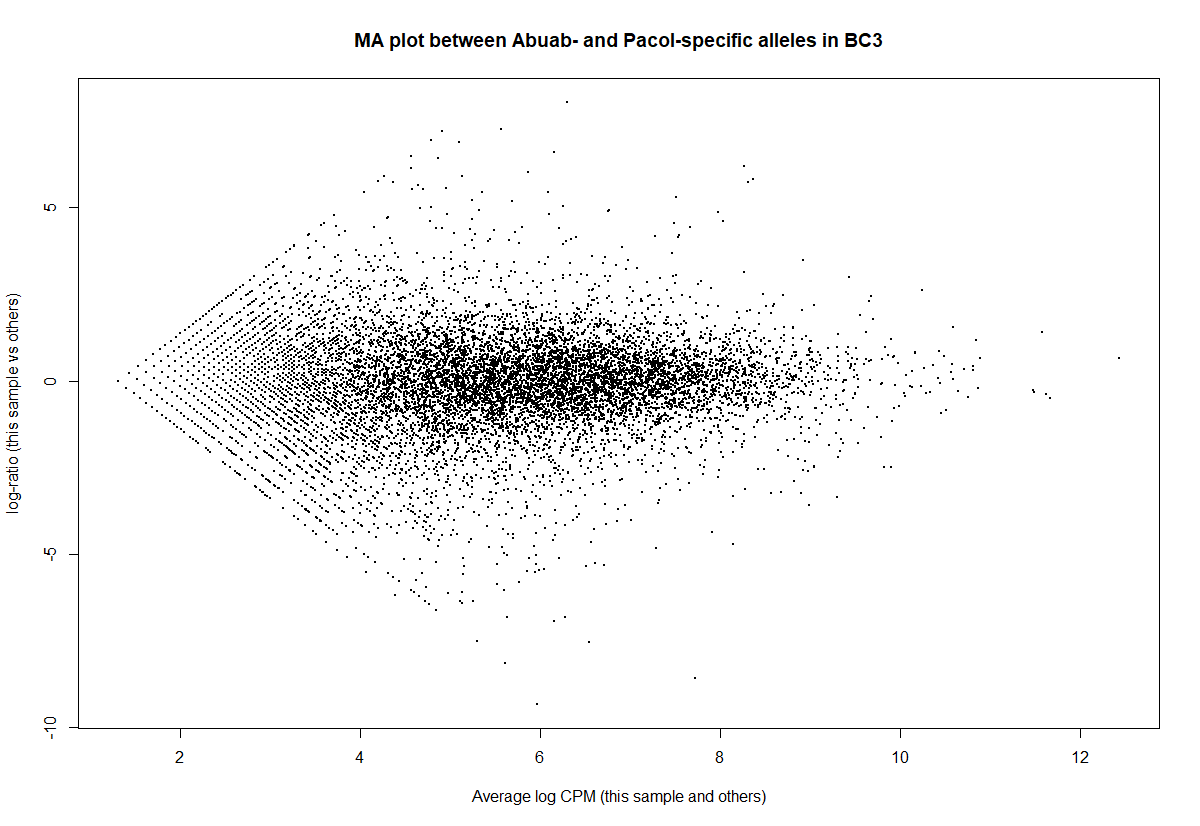

Supplement: Supplementary file 1 [file genes-13-01396-s001.zip › Figure. S2. MA plot between Abuab and Pacol specific alleles in BC3.png]
